# Comparison of Strength Adaptation Responses Among Individuals with Different Muscle Fiber Type Profiles

**DOI:** 10.3390/medicina62061069

**Published:** 2026-06-01

**Authors:** Enes Beltekin, Yunus Berk, İhsan Kuyulu, Dan Iulian Alexe, Teodora Isac, Gabriel Stănică Lupu, Răzvan Andrei Tomozei, Alina Elena Plasiciuc Ciobanu

**Affiliations:** 1 Department of Sports Management, Faculty of Sport Sciences, Bingol University, 12000 Bingol, Türkiye; ebeltekin@bingol.edu.tr; 2 Department of Coaching Education, Faculty of Sport Sciences, Van Yüzüncü Yıl University, 65080 Van, Türkiye; yunusberk@yyu.edu.tr; 3 Department of Recreation, Faculty of Sport Sciences, Bingol University, 12000 Bingol, Türkiye; ikuyulu@bingol.edu.tr; 4 Department of Physical and Occupational Therapy, “Vasile Alecsandri” University of Bacau, 600115 Bacău, Romania; 5 Faculty of Medicine, University of Medicine and Pharmacy “Carol Davila”, 050474 Bucharest, Romania; teodora.isac@umfcd.ro; 6 Fundeni Clinical Institute, 022328 Bucharest, Romania; 7 Department of Physical Education and Sport, “Alexandru Ioan Cuza” University of Iași, 700506 Iași, Romania; andrei.tomozei@uaic.ro; 8 Department of Health and Motricity, “Constantin Brâncuși” University of Târgu Jiu, 210185 Târgu Jiu, Romania; elenne10@yahoo.com

**Keywords:** muscle fiber type, resistance training, strength development, 1RM, fitness and bodybuilding

## Abstract

*Background and Objectives*: Muscle hypertrophy and strength increase are significantly influenced by the distribution of muscle fiber types in individuals. Individuals exposed to similar training intensity in fitness and bodybuilding exhibit different levels of adaptation suggesting that muscle fiber type may influence training outcomes. The aim of this study is to comparatively examine the strength development that occurs in fitness and bodybuilding athletes with different muscle fiber types (Type I, Type II, and Mixed) following a standardized resistance training program. *Materials and Methods*: The study was conducted using a quasi-experimental design based on a single-group pre-test–post-test model, with no control group. A total of 48 male athletes aged 19–26 years (22.75 ± 1.68) who had been regularly training in fitness and bodybuilding for at least two years voluntarily participated in the study. Muscle fiber types of the participants were indirectly estimated based on the number of repetitions performed at 80% of one-repetition maximum (1RM) in the bench press exercise, and individuals were divided into three groups: Type I, Type II, and mixed. All participants underwent a resistance training program for 6 weeks, 3 days a week, at 80% 1RM intensity and in the range of 8–12 repetitions. Data were analyzed using Shapiro–Wilk normality test, Wilcoxon Signed-Rank Test, and Mann–Whitney U. *Results*: The findings showed statistically significant increases between pre-test and post-test measurements in all groups. When percentage improvements were examined, the highest increase was observed in individuals with Type II muscle fiber type, and the lowest increase was observed in individuals with Type I muscle fiber type. *Conclusions*: In conclusion, the findings indicate that resistance training increases strength development in all muscle fiber types, but the level of development differs depending on the muscle fiber type. These findings highlight the importance of training programs based on individual muscle fiber type characteristics.

## 1. Introduction

Muscle hypertrophy and strength adaptations are primary physiological responses induced by resistance training. Muscle hypertrophy refers to the increase in the cross-sectional area and volume of skeletal muscle resulting from a positive muscle protein balance. Strength adaptations involve both neural and morphological mechanisms that enhance force-producing capacity [1,2]. Hypertrophic adaptations resulting from resistance training are primarily explained by myofibrillar and sarcoplasmic adaptations. Myofibrillar hypertrophy refers to the growth of contractile structures through an increase in actin and myosin filaments, while sarcoplasmic hypertrophy encompasses the volumetric increase in non-contractile components (glycogen, water, and mineral content) in the muscle cell [3].

Although the strength adaptation mechanism is based on physiologically similar principles, significant differences in developmental levels are observed among individuals. A significant portion of these differences are related to genetic factors, and it is reported that approximately 30–80% of muscle strength and hypertrophy potential are influenced by heritable traits [4]. Inter-individual variability in muscle strength and hypertrophic responses appears to be strongly influenced by genetic predisposition, with previous evidence indicating heritability estimates ranging from approximately 30% to 85% for skeletal muscle phenotypes [5]. Similarly, skeletal muscle fiber type distribution has also been shown to have a significant genetic determination [6].

Skeletal muscle consists of a heterogeneous combination of muscle fibers with different metabolic and functional characteristics. Muscle fibers are generally classified as slow-twitch Type I and fast-twitch Type II fibers [7]. Type I fibers are based on oxidative metabolism and exhibit high fatigue resistance during prolonged, low-intensity activities, while Type II fibers are based on anaerobic energy production and have a higher capacity to generate force and power [8]. Muscle fiber distribution in humans shows wide variation among individuals and can affect performance characteristics and adaptive response to training [9]. Furthermore, the higher carnosine concentration in fast-twitch fibers provides a significant physiological advantage in terms of high-intensity exercise performance [10,11].

Muscle fibers exhibit adaptations specific to the type of training stimulus they are exposed to [12]. High mechanical stress promotes Type II fiber adaptations, while prolonged metabolic stress is more closely associated with the development of Type I fibers. Therefore, determining the dominant muscle fiber type in individuals is considered an important parameter for individualizing training programs.

While direct methods like biopsy and molecular analysis provide high accuracy in determining muscle fiber type, their invasiveness and application difficulties limit their practical use. Therefore, more accessible indirect methods are widely preferred in sports science. Among indirect methods, physical performance tests, based on the number of repetitions an individual performs at a specific percentage load, are among the most frequently used approaches [13].

Although the physiological mechanisms determining strength adaptation are widely defined, differences in adaptation depending on muscle fiber type in individuals subjected to equal training loads remain a subject of debate. While a significant portion of current studies have examined muscle fiber characteristics in terms of performance differences, research comparing developmental responses under standardized loading conditions, particularly in fitness and bodybuilding athletes, is limited. This makes it difficult to clearly establish the true effect of muscle fiber type on hypertrophic adaptation.

In this context, determining whether an individual’s dominant muscle fiber type alters their developmental response to the same training stimulus can offer significant scientific and practical implications for individualizing training programs. Therefore, the aim of this study is to comparatively examine the strength development that occurs in fitness and bodybuilding athletes with different muscle fiber types (Type I, Type II, and Mixed) following a standardized resistance training program, as described in the abstract.

While muscle biopsy is considered the gold standard method for directly determining muscle fiber type, indirect methods are frequently preferred in sports science research due to their difficulty of application, invasive nature, and limitations in field conditions [14]. It is reported that performance tests based on the number of repetitions, which can be performed at a specific percentage load, can provide functional information about individuals’ muscle fiber composition and offer predictions regarding the dominance of fast or slow-twitch fibers [15]. Accordingly, athletes were divided into three groups based on the determined performance criteria: individuals with Type I, Type II, and Mixed fiber types.

The primary aim of the study was not to evaluate the effectiveness of resistance training itself, which is already well established in the literature, but rather to compare inter-individual adaptive responses under identical loading conditions. For this reason, a within-subject pre–post design combined with between-group comparisons was considered appropriate to isolate differences related to muscle fiber typology.

This study hypothesizes that individuals with a predominance of fast-twitch muscle fibers will show greater strength and hypertrophy development compared to individuals with slow-twitch muscle fibers under the same resistance training protocol.

## 2. Materials and Methods

### 2.1. Study Design

This study employs a quasi-experimental research design aimed at examining the effect of a resistance training program applied to a single study group using pre-test and post-test approach. Pre-test and post-test measurements were taken from participants before and after training. All participants received the same training intervention, without randomization or a control group. However, following baseline testing, subjects were subsequently classified according to their dominant muscle fiber type, and both within-group and between-group variations were analyzed. This design was preferred because it allows for direct comparison of the effect of the applied training protocol on the dependent variable [16]. This study was conducted at the Fitness and Bodybuilding Center of Van Yüzüncü Yıl University.

### 2.2. Subjects

**Figure 1:**
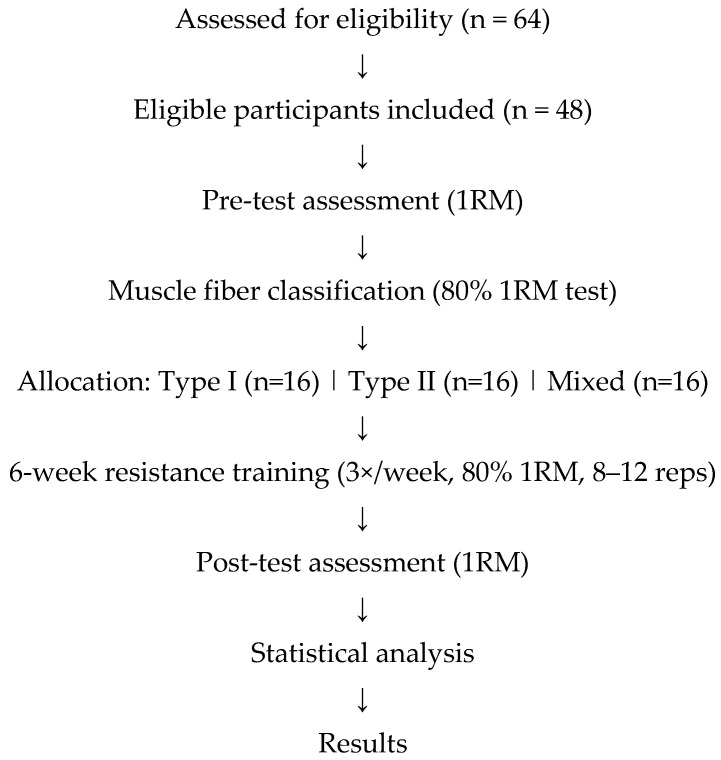
Study Flow Diagram.

The study group consisted of 48 male fitness and bodybuilding athletes who voluntarily participated in the study. Subjects were individuals aged 19–26 (22.75 ± 1.68), with heights between 167 and 186 cm (178.32 ± 5.55), weights between 60 and 108 kg (82.06 ± 10.42), and who had been regularly training in fitness and bodybuilding for at least two and at most five years, to ensure that individuals included in the study possessed the technical competence to perform the movements. Participants were instructed to adhere to the specified days and times throughout the study period, maintain their usual dietary habits, and refrain from participating in activities involving physical activity. All procedures were conducted in accordance with ethical principles of Helsinki [17]. Ethical Approval for the present study was granted by the Ethics Committee (2026/02-19, Van Yüzüncü Yıl University Social and Human Sciences Publication Ethics Committee). The experimental design and participant flow are illustrated in Figure 1. 

### 2.3. Inclusion and Exclusion Criteria

Participants were excluded from the study if they met any of the following criteria:

• Having a history of musculoskeletal injury, surgical intervention, or ongoing rehabilitation within the previous six months;
• Presence of any cardiovascular, metabolic, neurological, or orthopedic disorder that could influence physical performance or muscle metabolism;
• Regular use of ergogenic aids, anabolic agents, hormonal supplements, or performance-enhancing medications during the study period;
• Failure to comply with the scheduled testing sessions, training protocol, or measurement procedures;
• Participation in additional structured resistance training programs, high-intensity physical activities, or other competitive sports outside the study protocol;
• Inability to perform the required exercises with adequate technical proficiency or situations compromising testing safety;
• Withdrawal of voluntary consent at any stage of the research process.

Participants were considered drop-outs and excluded from the final analysis if they:

• Missed more than 20% of the scheduled training or testing sessions;
• Were unable to continue due to injury, illness, or medical conditions occurring during the study period;
• Participated in additional training programs or high-intensity physical activities outside the experimental protocol;
• Reported substantial changes in dietary habits or lifestyle behaviors inconsistent with study instructions;
• Failed to complete measurement procedures or compromised testing safety;
• Voluntarily withdrew from the study at any stage.

### 2.4. Measurement Procedures

Pre-test and post-test measurements were conducted to evaluate the training effect intervention. Participants refrained from strenuous physical activity for 48 h before testing.

To determine maximum capacity and observe the effect of the training on capacity, direct 1RM measurements were taken before and after the bench press exercise. Before the 1RM measurement and before all training sessions, participants performed a 10 min general warm-up, an 8 min dynamic warm-up, and a 5 min chest warm-up [18,19,20].

For all athletes in the groups, one-repetition maximum (1RM) measurements were taken using bench press in the chest muscle region. Direct measurements were not taken for exercises targeting other muscle groups included in the training program; loads were determined through observational assessments by experienced fitness and bodybuilding experts to ensure participants reached exhaustion in the 8–12 repetition range [21].

The primary muscles involved in the bench press exercise are the pectoralis major, triceps brachii, and anterior deltoid. The muscles that act as synergists and stabilizers are the serratus anterior, rotator cuff, latissimus dorsi, rectus abdominis, and biceps brachii [22,23,24]. Because skeletal muscles located in different regions of the human body play a role in the movement and these muscles have different muscle fiber type distributions, the bench press exercise was used to objectively evaluate the response to training.

The bench press exercise involves the integrated contribution of several prime mover, synergist, and stabilizer muscles with varying muscle fiber type compositions, making it a reliable multi-joint exercise for assessing maximal strength performance and neuromuscular adaptations to resistance training [21,22,23,24,25].

### 2.5. 1RM Testing Protocol

The one-repetition maximum (1RM) was performed according to the procedure established by [26]. A warm-up set of five repetitions was performed at 50% of the participant’s estimated 1RM, followed by four repetitions at 80% of the estimated 1RM. This was followed by a single repetition at 90% of the estimated 1RM (Table 1). Then, sets were performed with increasing load in small increments (10%) until failure was reached. Complete rest was performed between sets. To minimize fatigue and ensure maximum performance, 1RM was generally reached near maximum or with maximum load on the 3rd or 4th attempt for all participants [26].

**Table 1:** 1RM determination protocol.

%50	%80	%90	10% Increase
5 repetitions	4 repetitions	1 repetitions	failure

### 2.6. Muscle Fiber Type Determination

Muscle fiber typology was indirectly estimated using a repetition-based performance test at 80% of 1RM [27]. According to this method, if fewer than 8 repetitions are performed 80% of the 1RM, Type II fast-twitch muscle fibers are more dense; if 9–10 repetitions are performed, mixed muscle fibers, i.e., an equal distribution, are present; and if more than 11 repetitions are performed, Type I slow-twitch muscle fibers are denser [28]. 80% of the 1RM is considered the most suitable load for indirectly estimating muscle fiber composition (Table 2).

**Table 2:** Muscle fiber type determination protocol.

%80 1RM		
8 or less	9–10	11 or more
Type II	Mixed	Type I

It should be acknowledged that the present classification does not represent a direct histological determination of muscle fiber composition. Instead, the repetition-based assessment performed at 80% of the 1RM provides a functional and performance-oriented estimation of muscle fiber typology. Previous studies have suggested that repetition capacity at relative loads reflects underlying neuromuscular and metabolic characteristics associated with fiber-type dominance rather than absolute fiber distribution. Therefore, the classification used in this study should be interpreted as a phenotypic proxy of muscle fiber typology rather than a direct physiological measurement.

### 2.7. Training Intervention

Participants underwent muscle resistance training every other day, 3 days a week, for 6 weeks. Training intensity was determined by taking 80% of the individual’s 1RM. The number of repetitions was 8–12, with a 2 min rest between sets. No increase in load was made during the training process; the determined program was applied for 6 weeks (Table 3).

**Table 3:** Fitness and bodybuilding training program.

Monday		Wednesday		Friday	
Bench Press		Biceps Curl		Triceps Pushdown	
4 Set	8–12 repetitions	4 Set	8–12 repetitions	4 Set	8–12 repetitions
Pec Deck Fly		Hammer Curl		Reverse Pushdown	
4 Set	8–12 repetitions	4 Set	8–12 repetitions	4 Set	8–12 repetitions
Leg Ekstension		Crunch		Seated Row	
4 Set	8–12 repetitions	Body Weight	20 repetitions	4 Set	8–12 repetitions
Leg Curl		Leg Raise		Lat Pulldown	
4 Set	8–12 repetitions	Body Weight	20 repetitions	4 Set	8–12 repetitions
Training Intensity		Rest between Sets		Training Duration	
%80		2 Minutes		40–45 Minutes	

Training loads were intentionally kept constant throughout the intervention period to maintain a standardized mechanical stimulus across participants. This approach was preferred to minimize confounding adaptations resulting from individualized load progression and to allow clearer comparison of training responses between different muscle fiber typology groups.

### 2.8. Statistical Analysis

The data obtained from the study were analyzed using the SPSS 25 software package. The normality assumption of the data was evaluated with the Shapiro–Wilk test, and it was determined that the pre-test (W = 0.914; *p* = 0.002) and post-test (W = 0.907; *p* = 0.001) variables did not show a normal distribution. Therefore, non-parametric statistical methods were preferred in the analyses. Within-group pre-test–post-test changes were examined with the Wilcoxon Signed Ranks test. The Kruskal–Wallis test was used to determine the differences between groups with three different muscle fiber types. In cases where a significant difference was found in the Kruskal–Wallis analysis, pairwise comparisons between groups were performed as post hoc analysis with the Mann–Whitney U test.

In the analyses, performance changes were evaluated based on absolute measurement values; percentage change values were reported only in descriptive analyses to show the relative magnitude of individual improvement. Statistical tests were applied using raw measurement values instead of percentage conversions.

A post hoc power analysis indicated that the achieved sample size provided sufficient statistical power (>0.80) to detect moderate-to-large effects between groups, supporting the adequacy of the sample for the performed analyses.

## 3. Results

This section presents the statistical results of the study. It includes statistical analyses of the study participants based on various variables.

The individuals who voluntarily participated in the study were between 19 and 26 years old. Their height ranged from 167 to 186 cm. The athletes participating in the study weighed between 60 and 108 kg (Table 4 and Table 5).

**Table 4:** Distribution of participants’ age, height, and weight.

Variable	Mean ± SD	Min	Max
Age (years)	22.75 ± 1.68	19	26
Height (cm)	178.32 ± 5.55	167	186
Body Mass (kg)	82.06 ± 10.42	60	108

Values are presented as mean ± standard deviation (SD). N = 48.

**Table 5:** Sports experience distribution.

Sports Experience	N	Percent (%)	Cumulative (%)
2	2	4.2	4.2
3	23	47.9	52.1
4	16	33.3	85.4
5	7	14.6	100.0
**Total**	**48**	**100.0**	

The Shapiro–Wilk normality test revealed that both pre-test (W = 0.914, *p* = 0.002) and post-test (W = 0.907, *p* = 0.001) scores significantly deviated from normal distribution. Accordingly, non-parametric statistical methods were used in subsequent analyses (Table 6).

**Table 6:** Test of normality.

Variable	N	W	*p*
Pre-Test	48	0.914	0.002
Post-Test	48	0.907	0.001

The average 1RM (One Repetition Maximum) of the participants was 108.54 kg at the initial measurement and 119.23 kg at the final measurement, resulting in a difference of 10.69 kg. 80% of the initial 1RM value was 86.83 kg, and 80% of the final 1RM value was 95.38 kg, resulting in a difference of 8.550 kg. A significant increase was observed between pre-test and post-test values (Z = −6.054, *p* < 0.001). Effect size analysis showed that the implemented training protocol had a very large effect on performance (r = 0.87). A statistically significant difference was found between the 1RM measured before the training program (108.54 kg) and the 1RM measured after the training program (119.23 kg) (*p* < 0.001) (Table 7).

**Table 7:** Pre-test and post-test comparison using the Wilcoxon Signed Ranks Test.

Variable	N	Median (Q1–Q3)	Mean ± SD	Min–Max	Z	*p*	Effect Size (r)
First Maximal (Pre-Test)	48	100 (90–125)	108.54 ± 25.30	74–180	−6.054	<0.001	0.87
Second Maximal (Post-Test)	48	114 (98–132.75)	119.23 ± 25.54	80–200			

**Note.** Values are presented as median (Q1–Q3) and mean ± standard deviation (SD). Pre–post comparisons were performed using the Wilcoxon Signed Ranks Test. Effect size (r) was calculated as Z/√N and interpreted according to Cohen’s criteria.

Analysis results showed that there was a statistically significant difference between the post-test and pre-test among the groups (χ^2^(2) = 32.789; *p* < 0.001). When the mean ranks were examined, it was determined that the highest value was in the Type II group (Table 8).

**Table 8:** Intergroup pre-test post-test comparison.

Variable	Group	N	Mean Rank	H (χ^2^)	*p*
Post-Test Pre-Test Difference	Type I	16	12.88	32.789	<0.001
	Type II	16	39.38		
	Mixed	16	20.03		

According to the analysis results, a statistically significant difference was found between Type I and Type II groups. When the average rankings were examined, it was determined that the Type II group had higher values. A statistically significant difference was also found between Type I and the Mixed group, favoring the Mixed group. Furthermore, a statistically significant difference was found between Type II and the Mixed group, again favoring the Type II group. When mean values were examined, the highest growth occurred in those with Type II muscle fiber type, and the least growth occurred in those with Type I muscle fiber type (Table 9).

**Table 9:** Comparing differences between groups.

Variable	Group	N	Mean Rank	U	Z	*p*
İntergroup Difference	Type I	16	8.50	0.000	−4.889	0.001
	Type II	16	24.50			
	Type I	16	12.88	0.029	−2.301	0.021
	Mixed	16	20.13			
	Type II	16	23.88	0.000	−4.500	0.000
	Mixed	16	9.13			

*p* < 0.001.

**Figure 2:**
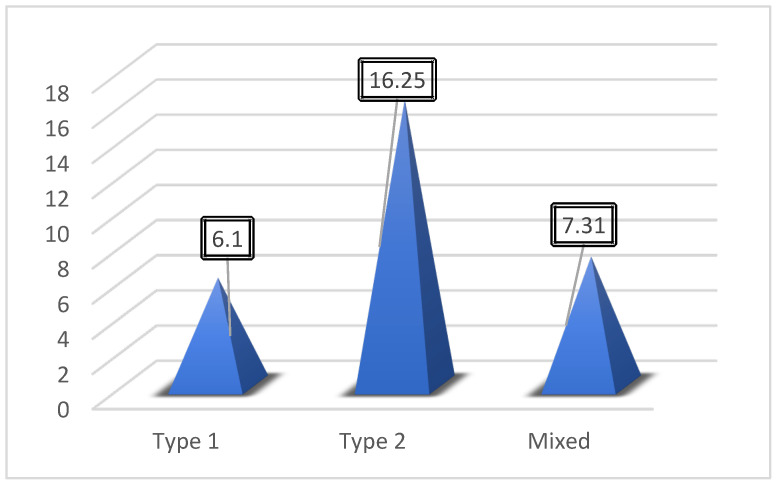
Growth rate according to muscle fiber type (%).

Pre-test–post-test comparisons showed increases of 6.10% (Type I), 16.25% (Type II), and 7.31% (Mixed), respectively. The highest improvement was observed in individuals with Type II muscle fiber type (Figure 2).

## 4. Discussion

The study investigated the effect of a resistance training protocol on maximal strength (1RM) in participants with different muscle fiber types. Results demonstrated a significant increase in 1RM following the training intervention, from 108.54 ± 25.30 (pre-test) to 119.23 ± 25.54 (post-test), with a very large effect size (r = 0.87).

When examining differences between fiber type groups, the Type II participants exhibited the largest increases in 1RM, followed by mixed group, and type I showed the smallest gain. Intergroup comparisons revealed statistically significant differences in strength gains between the Type I and Type II groups (*p* = 0.001), the Type I and mixed groups (*p* = 0.021), and the Type II and mixed groups (*p* < 0.001). This suggests that muscle fiber composition plays a notable role in the magnitude of strength improvements in response to resistance training, with a Type II advantage in maximal force production [29].

It is important to note that strength improvements observed during short-term resistance training interventions are partly attributed to neural adaptations, including improved motor unit recruitment, synchronization, and firing frequency [30,31].

Muscle composition and muscle fiber type are important determinants of differences in physical and physiological adaptations to training stimuli among individuals [31,32,33]. Type I (slow-twitch) fibers are better suited for prolonged submaximal contractions due to their high oxidative capacity, mitochondrial density, and fatigue resistance; while Type II fibers provide an advantage in strength and power-requiring activities thanks to their larger cross-sectional area, higher contraction speed, and anaerobic energy production capacity [7,32,34]. These physiological differences can lead to variable performance responses among individuals, even with the same training protocol [27].

Although muscle biopsy is considered the gold standard method for determining muscle fiber composition, indirect methods are more frequently preferred in field studies due to its invasive nature and application difficulties [28,35]. Literature shows that the number of repetitions performed at a specific 1RM percentage can provide functional information about muscle fiber distribution, and that inter-individual repetition differences are related to fiber type heterogeneity [27,36]. Indeed, studies conducted at 80–85% 1RM loads have shown that individuals with a high proportion of slow-twitch fibers can perform more repetitions, while individuals with a predominance of fast-twitch fibers reach exhaustion earlier [28,37,38]. Similarly, it has been reported that individuals with a high density of fast-twitch fibers experience faster on-set fatigue and may have longer recovery times [39,40].

Metabolic characteristics are at the root of performance differences depending on muscle fiber type. Fast-twitch fibers have a higher rate of ATP consumption and are more dependent on glycolytic metabolism, which can lead to faster accumulation of metabolic byproducts and earlier onset of peripheral fatigue [41,42,43]. In contrast, Type I fibers can maintain energy production throughout increased set duration, thus providing an endurance advantage at moderate loads.

The observed difference in repetition performance among individuals under the same relative load and the earlier onset of exhaustion in individuals with a predominance of fast-twitch fibers are consistent with previous studies reporting that muscle fiber composition affects performance [28,37,38]. Similarly, it has been reported that metabolic fatigue develops faster and repetition capacity may be limited in individuals with a high proportion of fast-twitch fibers [38,39]. However, the wide individual variation in the number of repetitions under the same relative load indicates that it is not solely dependent on muscle fiber type; training history, technical proficiency, motivation level, and neural adaptations also play significant roles [13].

The fact that a significant portion of strength increases, particularly in short-term resistance training, can be explained by neural adaptations [44] limits the attribution of performance improvement solely to muscle fiber structure. Therefore, while current findings support the idea that muscle fiber type may influence repetition performance, it should be considered that performance adaptation is a multifactorial process. The larger improvements observed in Type II dominant individuals may be explained by greater recruitment of high-threshold motor units under high-load resistance training.

In light of this information, it may not be sufficient to explain the strength increases observed in the present study solely through hypertrophic adaptations dependent on muscle fiber type. It is known that a significant portion of early adaptations to resistance training stem from neural adaptation mechanisms such as motor unit synchronization, increased firing frequency, and decreased antagonist muscle activation [44]. It should be considered that strength gains, especially in short-term interventions, may be related to neural adaptations rather than morphological changes. This is an important factor to consider when interpreting the performance improvements observed in this study.

In conclusion, while direct measurement methods provide higher accuracy, indirect methods may offer a practical alternative, especially for amateur athletes and in fitness applications. However, considering individual physiological differences instead of applying the same load and repetition schedule to all individuals in training planning can contribute to achieving safer and more effective adaptations.

## 5. Conclusions

This quasi-experimental study included a total of 48 volunteer athletes aged 19–26 years, with heights of 167–186 cm and body weights of 60–108 kg, who had been regularly training in fitness and bodybuilding for at least two years. To functionally classify muscle fiber types, 1-repetition maximum (1RM) measurements were taken, and an indirect muscle fiber type determination method based on physical performance was applied using 80% of the 1RM. As a result of this evaluation, three equal groups (n = 16) were formed showing Type I, Mixed, and Type II muscle fiber characteristics. All participants underwent a standardized hypertrophy-focused training program with a training intensity of 80% 1RM and repetitions ranging from 8 to 12. Following pre-test measurements, the training intervention lasted six weeks, three days a week, and then post-test measurements were taken.

When comparing pre-test and post-test measurements, a statistically significant increase in the total weight lifted was determined. This finding suggests that the implemented training program may be associated with performance improvement in all muscle fiber types. When examining the intergroup variation, it was observed that the relative improvement was higher in participants exhibiting Type II muscle fiber characteristics and relatively more limited in individuals with predominantly Type I characteristics. However, due to the lack of a control group in the study design and the indirect determination of muscle fiber type, it is not possible to attribute the observed differences solely to muscle fiber composition. Therefore, the findings suggest that muscle fiber type may be one of the factors influencing training response, but do not allow for definitive generalizations regarding individual performance improvement.

### Limitations of the Study

The study design has some limitations. First, the research does not include a randomized controlled experimental design, and the absence of a control group limits the attribution of the observed changes solely to the applied training protocol. Furthermore, muscle fiber type was determined through indirect performance tests rather than direct biopsy. While indirect methods offer practical advantages, they provide a functional prediction rather than a precise determination of fiber type. In addition, variables such as individuals’ training history, technical skill level, and motivation are among the potential confounding factors that may affect repetition performance [13].

Literature review generally shows that the vastus lateralis muscle is considered for determining muscle fiber type. From a different perspective, in this study, the pectoral muscles of the upper extremity were used to determine muscle fiber type distribution. This may have resulted in the effectiveness and growth rate of type II muscle fibers being higher than those of type I and mixed muscle fibers.

The lack of sufficient scientific literature comparing non-invasive performance tests with direct measurement methods reduces the likelihood that physical performance tests are a scientifically proven method.

The lack of established standards for physical performance tests found in the literature, and the fact that measurements are taken for different muscle groups, at different loads and repetition numbers, weakens the reliability of these tests.

The lack of studies comparing indirect measurement methods used in muscle fiber type determination with direct measurement methods poses a limitation in terms of validity and reliability.

Another limitation is that muscle fiber typology was estimated using an indirect performance-based method rather than muscle biopsy. While this approach increases ecological validity and practical applicability, it may introduce classification variability. Future studies combining non-invasive performance assessments with direct physiological measurements are warranted.

## Data Availability

The raw data supporting the conclusions of this article will be made available by the authors, without undue reservation.
